# Effect of Various Type of Nanoparticles on Mechanical and Tribological Properties of Wear-Resistant PEEK + PTFE-Based Composites

**DOI:** 10.3390/ma14051113

**Published:** 2021-02-27

**Authors:** Sergey V. Panin, Duc A. Nguyen, Dmitry G. Buslovich, Vladislav O. Alexenko, Aleksander V. Pervikov, Lyudmila A. Kornienko, Filippo Berto

**Affiliations:** 1 Laboratory of Mechanics of Polymer Composite Materials, Institute of Strength Physics and Materials Science SB RAS, 634055 Tomsk, Russia; buslovichdg@gmail.com (D.G.B.); vl.aleksenko@mail.ru (V.O.A.); rosmc@ispms.ru (L.A.K.); 2 Department of Materials Science, Engineering School of Advanced Manufacturing Technologies, National Research Tomsk Polytechnic University, 634030 Tomsk, Russia; 3 Seaside Branch Russian-Vietnamese Tropical Center, Department of Tropical Endurance, Nha Trang 57106, Vietnam; nda.ttndvn@gmail.com; 4 Laboratory of Physical Chemistry of Ultrafine Materials, Institute of Strength Physics and Materials Science SB RAS, 634055 Tomsk, Russia; pervikov@list.ru; 5 Faculty of Engineering, Department of Mechanical and Industrial Engineering, Norwegian University of Science and Technology, 7491 Trondheim, Norway; filippo.berto@ntnu.no

**Keywords:** polyetheretherketone (PEEK), nanoparticles, solid lubricant fillers, wear factor, transfer film

## Abstract

The mechanical and tribological properties of polyetheretherketone (PEEK)- and PEEK + PTFE (polytetrafluoroethylene)-based composites loaded with and four types of nanoparticles (carbonaceous, metallic, bimetal oxide, and ceramic) under metal- and ceramic-polymer tribological contact conditions were investigated. It was found that loading with the nanofillers in a small content (0.3 wt.%) enabled improvement of the elastic modulus of the PEEK-based composites by 10–15%. In the metal–polymer tribological contact, wear resistance of all nanocomposites was increased by 1.5–2.3 times. In the ceramic-polymer tribological contact, loading PEEK with metal nanoparticles caused the intensification of oxidation processes, the microabrasive counterpart wear, and a multiple increase in the wear rate of the composites. The three component “PEEK/10PTFE/0.3 nanofillers” composites provided an increase in wear resistance, up to 22 times, for the metal–polymer tribological contact and up to 12 times for the ceramic-polymer one (with a slight decrease in the mechanical properties) compared to that of neat PEEK. In all cases, this was achieved by the polymer transfer film formation and adherence on the counterparts. The various effects of the four types of nanoparticles on wear resistance were determined by their ability to fix the PTFE-containing transfer film on the counterpart surfaces.

## 1. Introduction

Solid lubricants (primarily in the form of microparticles) are widely used for friction units, especially containing polymer composites. The most common are polytetrafluoroethylene (PTFE), graphite [1], molybdenum dioxide (MoS_2_) [2], boron nitride (BN), and some others [3].

PTFE is one of the best fillers to improve wear resistance of polyetheretherketone (PEEK)-based composites. Lu and Friedrich [4] investigated the tribological properties of the PEEK-based composites loaded with PTFE. Dry sliding friction tests were carried out according to the “pin-on-disk” scheme at a sliding speed of 1 m/s, and a contact pressure of 1 MPa. It was shown that loading with PTFE enabled significant reduction of the friction coefficient and wear rate. In this case, the optimum PTFE content ranged from 10 to 20 vol.% (23 to 45 wt.%). It was found that a multiple reduction in the friction coefficient and wear rate was achieved due to the formation of a transfer film on the surface of a steel counterpart.

The effect of decreasing both the friction coefficient and wear rate of the PEEK-based composites loaded with PTFE is described in various papers [5,6,7]. However, the optimum PTFE content varied greatly. For example, Bijwe et al. [6] showed that the maximum wear resistance (30 times higher than that for neat PEEK) was achieved by loading with 7.5 wt.% PTFE. In another case [8], the “70 wt.% PEEK + 30 wt.% PTFE” composite possessed the highest tribological properties (its wear resistance increased by 900 times compared to that of neat PEEK, and 260,000 times compared to that of neat PTFE). It is possible that this distinction was related to the test conditions (namely reciprocating movement or the “pin-on-disk” scheme, the *P·V* (load sliding speed) factor, etc.), as well as the composite manufacturing methods.

Hufenbach et al. [5] showed a decrease in the mechanical properties of the PEEK-based composites loaded with PTFE, which might limit their use under severe loading conditions. To compensate for this drawback, other fillers were added into the composites, for example, reinforcing fibers or particles, etc. This approach was applied in various studies [9,10,11]. Xie et al. [9] used potassium titanate (K_2_TiO_3_) to improve the mechanical and tribological properties of the PEEK-based composites loaded with PTFE. It was shown that loading with K_2_TiO_3_ enabled increase of both, microhardness and wear resistance. In the studied case, the optimal K_2_TiO_3_ content was 5 wt.%. In another case [10], loading with graphene nanoparticles in an amount of 2 wt.% resulted in wear resistance enhancing of the “PEEK + 10 wt.% carbon fibers (CF) + 10 wt.% PTFE” composite.

Graphite possesses excellent lubricity under dry sliding friction conditions. Therefore, it is widely used to enhance the tribological properties of the PEEK-based composites. The results of the investigations [12,13,14] showed that graphite reduced the friction coefficient and increased wear resistance of the multicomponent PEEK-based composites. In addition, loading with graphite improved the heat dissipation characteristics of the polymer composites in some cases [15]. However, graphite is not efficient under the water lubrication conditions, since water adsorption reduces the binding energy between hexagonal planes, which resulted in its rapid abrasion due to friction [16].

The layered structure of molybdenum disulfide (MoS_2_) determines its unique antifriction properties and enables its efficient application as a solid lubricant [17,18,19,20,21,22,23]. Zalaznik et al. [18] studied the effect of types, sizes, and contents of solid lubricant particles on the tribological properties of the PEEK-based composites. Two types of solid lubricant nano- and microparticles were investigated—molybdenum disulfide (MoS_2_) and tungsten disulfide (WS_2_), with contents from 0.5 to 5 wt.%. It was shown that solid lubricant particles at their maximum content of 5 wt.% could reduce the friction coefficient of the PEEK-based composites from 0.6 down to 0.4, regardless of the type and size. However, wear resistance was improved only in the case of loading with MoS_2_ micro- or nanoparticles or WS_2_ nanoparticles, in amounts of 1 wt.%. A further increase in the filler content reduced wear resistance of the PEEK-based composites.

Theiler and Gradt [24] compared the efficiency of MoS_2_ and graphite loaded in the anti-friction “PEEK + PTFE + CF” composites for friction in vacuum. It was shown that loading with MoS_2_ particles provided better wear resistance compared to graphite, especially at low temperatures (−80 °C). The low friction coefficient and high wear resistance was achieved due to the formation of a thin transfer film on the surface of a steel counterpart.

Many authors [25,26,27,28,29] also reported on the possibility of wear resistance improving of the polymer composites, by loading with solid microparticles like titanium dioxide (TiO_2_), zirconium dioxide (ZrO_2_), silicon carbide (SiC), copper oxide, and sulfide (CuO and CuS), etc. Bahadur et al. [25], the tribological properties of the PEEK-based composites loaded with CuO, CuS, and CuF_2_ microparticles in an amount of 35 vol.% was studied. It was shown that despite the high friction coefficient, the composites loaded with solid microparticles possessed better wear resistance compared to that of neat PEEK. Bahadur et al. [26,27,28,29] also confirmed the possibility of wear resistance increase of the polyphenylene sulfide (PPS)-based composites, by loading with solid microparticles.

Progress in the field of nanotechnologies intensified research on the effect of nanoscale fillers, on the performance properties of polymers and their composites. Compared to the micro-scale ones, the nanofillers possess some advantages—(a) less abrasive effect on counterparts; (b) a large specific surface area, and therefore, a large adhesion to the polymer matrix; and (c) the possibility of improving a number of the composite characteristics with a relatively low filler content. In addition, a chance to increase the tribological properties of the composites based on the promising polymers by loading with nanoparticles is discussed [30,31,32,33,34].

Wang et al. [30] investigated the PEEK-based composites loaded with Si_3_N_4_ nanoparticles, obtained by compression sintering. Wear tests were carried out according to the “block-on-ring” scheme. It was found that the composite containing 2.8 vol.% Si_3_N_4_ possessed the lowest wear rate. The PEEK-based nanocomposites loaded with other nanoparticles (SiO_2_ and ZrO_2_) were also studied [31,32]. It was shown that the lowest wear rate was also obtained by loading with ~1.5–3.5 vol.% SiO_2_ and ZrO_2_ nanoparticles.

Bahadur S. et al. [33,34] tested the PPS-based nanocomposites loaded with Al_2_O_3_, TiO_2_, ZnO, CuO, and SiC particles, according to the “pin-on-disk” scheme. It was shown that the nanoparticle content of 2 vol.% was the optimum for the designed wear-resistant PPS-based nanocomposites. Table 1 presents compositions of some PEEK nanocomposites [35]. The maximum wear resistance was achieved by loading with ~7 wt.% nanoparticles.

Recently, carbon nanofibers (CNF) and carbon nanotubes were widely applied to design PEEK-based nanocomposites [36,37,38]. Werner P. et al. [36,37] showed that loading with CNF in amounts up to 15 wt.% enabled improvement of the elastic modulus of the PEEK-based composites. At the optimal CNF content (~10 wt.%), the wear rate of the nanocomposites decreased significantly compared to that of the neat PEEK.

In addition to the microfillers, the nano- ones were also loaded to design multicomponent composites, where each filler performed its own function [39,40,41,42]. Molazemhosseini et al. [39] studied the tribological properties of the PEEK-based composites loaded with CF and SiO_2_ nanoparticles. It was shown that at a content of 20 vol.% CF and 1.4 vol.% nano-SiO_2_, the friction coefficient and wear rate decreased under all tribological testing conditions. According to the authors, loading with nanoparticles could increase the strength at the interface between the CF and the polymer matrix, which improved wear resistance of the multicomponent PEEK-based nanocomposites.

Guo L. et al. [40] showed that loading with SiO_2_ nanoparticles in amounts up to 4 vol.% resulted in significant wear resistance improvement and reduction in the friction coefficient of the multicomponent PEEK-based composites, in comparison with the cases of loading with CF or PTFE, separately. The formation of a transfer film containing SiO_2_ nanoparticles was revealed, which was the main reason for the nanocomposite wear resistance improvement.

Thus, various types of fillers were applied to increase the wear resistance of the polymer-based composites. Each filler played its own role. By understanding their function in the composites, it becomes possible to design ones possessing the optimal properties, in accordance with specified operating conditions. 

In these investigations, the task was to study step-by-step, the effect of several types of nanofillers (carbonaceous, metallic, bimetal oxide, ceramic) on the mechanical and tribological properties of the PEEK-based composites, in order to design antifriction high-strength three-component ones for the operations in the metal- and ceramic-polymer tribological contacts of the friction units. Section 2 defines the used materials and the methods of their studies. The structure, the physical and mechanical properties of the “PEEK/0.3 nanoparticles” composites are presented in Section 3.1. Section 3.2 succinctly describes the mechanical and tribological properties of the “PEEK/7 nanofiller” composites, since this number of nanoparticles was the most widely studied previously. Section 3.3 was devoted to a detailed analysis of the structure, and the physical and mechanical properties of the “PEEK/10PTFE/0.3 nanoparticles” composites. At the end of the presentation of the results, the reasons for the observed processes are discussed and conclusions about the prospects for the use of nanocomposites are drawn.

## 2. Materials and Methods

The “Victrex” PEEK powder (450 PF, Victrex PLC, Lancashire, UK) with an average particle size of 50 μm was used as a matrix resin, which was loaded onto PTFE polytetrafluoroethylene (particle size of 6–20 μm, F4-PN20 grade, “Ruflon” LLC, Perm, Russia), with four types of nanofillers.

**Figure 1:**
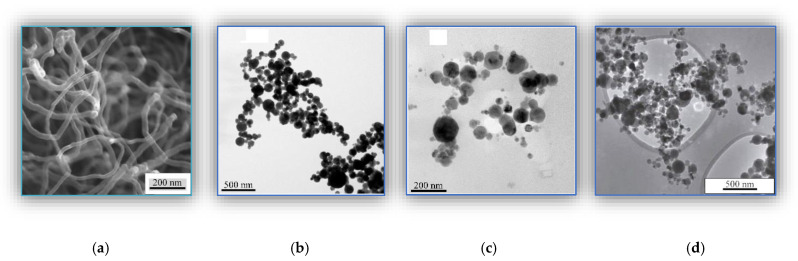
The TEM micrographs of nanofillers: (**a**) CNF; (**b**) Cu; (**c**) Fe-Cu-O; (**d**) SiO_2._

(1) Carbonaceous—the “Taunit” CNF (multiwall tubes) with an outer diameter of 60 nm and a length of 2–3 μm obtained by the gas-phase chemical deposition (NanoTechCenter LLC, Tambov, Russia), Figure 1a.
(2) Metallic—copper (Cu) nanoparticles with a size of 87 ± 1 nm, obtained by the exploding wire method (EWM) [43], Figure 1b.
(3) Bimetal oxide—copper ferrite (CuFe_2_O_4_) nanoparticles with a size of 34 ± 1 nm obtained by the exploding wire method (EWM), Figure 1c.
(4) Ceramic—the “Tarkosil” silicon dioxide (SiO_2_) nanoparticles with sizes of 25–35 nm fabricated by evaporating initial substances in an electron accelerator, Figure 1d.

Cu nanoparticles were produced in an argon atmosphere from the copper wires. Then, the nanoparticles were passivated to prevent their spontaneous combustion by slow air inlet into a chamber for 48 h. Fe-Cu-O nanoparticles were produced by electric exploding both iron and copper wires in an oxygen-containing atmosphere (Ar + 20 vol.% O_2_). The iron (66 at.%) to copper (34 at.%) ratio was determined by the wire diameters.

The average particle size was assessed by transmission electron microscopy (TEM). Figure 1 shows the TEM micrographs of the nanoparticles. Their parameters are given in Table 2.

Cylindrical workpieces were fabricated by compression sintering at a specific pressure of 15 MPa and a temperature of 400 °C. The subsequent cooling rate was 2 °C/min. The polymer powder and the fillers were mixed by dispersing the suspension components in alcohol using a “PSB-Gals 1335-05” ultrasonic cleaner (PSB-Gals, Ultrasonic equipment center, Moscow, Russia). The generator frequency and the processing duration were 22 kHz and 3 min, respectively. After mixing, the suspension was dried in an oven with forced ventilation at a temperature of 120 °C for 3 h. Using alcohol as a mixing medium suggested the absence of volatiles in the ready-made mixtures for hot pressing.

Samples were cut out from cylindrical blanks with the help of computer controlled Purelogic PLRA4 milling machine. The cutting speed was 100 m/min. Sample surfaces were free from visible cracks, scratches, and cavities. In addition, the sample surface was additionally polished with a sandpaper (grit P2000).

Shore D hardness was determined by an “Instron 902” facility (Instron, Norwood, MA, USA) in accordance with ASTMD 2240.

The tensile properties of the PEEK-based composite specimens were measured using an “Instron 5582” electromechanical testing machine (Instron). The specimen shapes met the requirements of ASTMD 638.

Wear tests were carried out according to the ʺpin-on-diskʺ scheme, under the dry sliding friction conditions, at a load of 10 N and a sliding speed of 0.3 m/s. A “CSEMCH-2000” tribometer (CSEM, Neuchâtel, Switzerland) was employed in accordance with ASTMG99. The diameter of counterparts was 6 mm. They were made from bearing steel with a hardness of HRC60 and ZrO_2_ ceramics. The sliding distance was 3 km and the tribological track radius was 10 mm, i.e., the rotation speed was 286 rpm.

The wear track profiles were determined using the data for at least 10 tracks. Then, the wear rate values were estimated on the basis of the experimental test data over at least four samples of each type. Mathematical statistics methods were used for the experimental results processing.

Surface topography of the wear tracks was studied using a “Neophot-2” optical microscope (Carl Zeiss, Oberkochen, Germany), equipped with a “Canon EOS 550D” digital camera (Canon Inc., Tokyo, Japan), and an “Alpha-Step IQ” contact profiler (KLA-Tencor, Milpitas, CA, USA).

A “Neophot 2” optical microscope (Carl Zeiss, Jenna, Germany) was used to examine the wear track surfaces after testing. The supermolecular structure of the composites was studied on the cleaved surfaces of the notched specimens, mechanically fractured after exposure in liquid nitrogen. A “LEO EVO50” scanning electron microscope (Carl Zeiss, Oberkochen, Germany) was employed at an accelerating voltage of 20 kV.

## 3. Results and Discussion

### 3.1. The PEEK-Based Composites Loaded with 0.3 wt.% Nanoparticles

Initially, the possibility of improving the mechanical and tribological properties of the PEEK-based composites by loading with nanoparticles of various types in an amount of 0.3 wt.% was studied. In order to compare the efficiency of loading with the nanofillers, four types were applied—(i) Carbonaceous—carbon nanofibers (CNF); (ii) Metallic—copper (Cu); (iii) Bimetal oxide—copper ferrite (CuFe_2_O_4_); and (iv) Ceramic—silicon dioxide (SiO_2_).

The physical and mechanical properties of neat PEEK and the PEEK-based composites are presented in Table 3. Loading PEEK with CNF did not cause a noticeable increase in tensile strength and the elastic modulus. Shore D hardness decreased slightly after loading with Cu metal particles. However, this parameter increased by one unit, after loading with SiO_2_ ceramic nanoparticles, which was highly likely, due to their higher hardness. The elastic modulus also increased by 10–15% after loading with all studied types of nanoparticles. After loading with SiO_2_ and CuFe_2_O_4_ particles, tensile strength was maintained at the neat PEEK level. Finally, elongation at break decreased by 5–10% for all types of the used nanoparticles.

**Figure 2:**
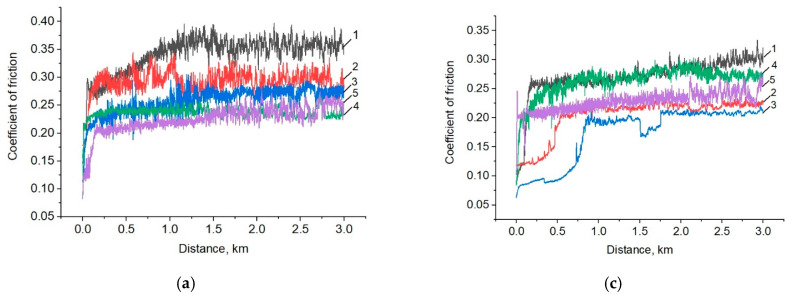

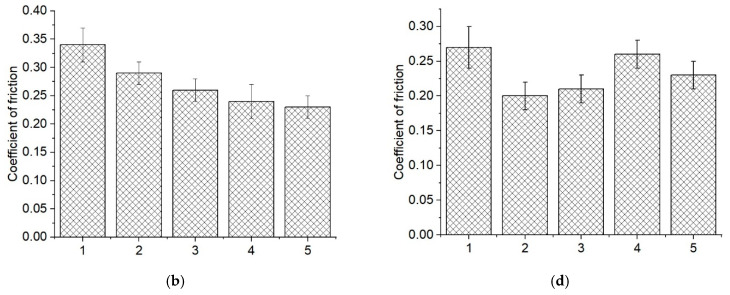
The dependences of the friction coefficients from the sliding distance (**a**,**c**), as well as their average values (**b**,**d**) for neat PEEK (1) and the PEEK-based nanocomposites loaded with 0.3 wt.% nanoparticles—CNF (2); Cu (3); CuFe_2_O_4_ (4); SiO_2_ (5); the metal (**a**,**b**) and ceramic (**c**,**d**) counterparts.

The tribological tests of the nanocomposites were carried out under the dry sliding friction conditions on the steel and ceramic counterparts. Figure 2 shows the dependence of the friction coefficients from the sliding distance, as well as their average values for neat PEEK and the PEEK-based nanocomposites. The friction coefficients of all studied nanocomposites decreased from 0.34 (for neat PEEK) down to 0.23–0.28 (the distinction was 35%), when sliding on the metal counterpart (Figure 2b). When sliding on the ceramic counterpart (Figure 2d), the “PEEK/0.3CNF” nanocomposite possessed the lowest friction coefficient. Its value was 0.20 ± 0.02, which was dozen percent less than that of neat PEEK. The friction coefficients of the composites loaded with CuFe_2_O_4_ bimetal oxide particles decreased insignificantly with respect to that of neat PEEK. According to the authors, a noticeable decrease in the friction coefficient by loading with CNF was associated with the type of the nanocomposite supermolecular structure formed (similar to neat PEEK one). Another reason was the formation of a transfer film (it is discussed below in the analysis of the wear track surfaces of both the polymer composites and the counterparts).

**Figure 3:**
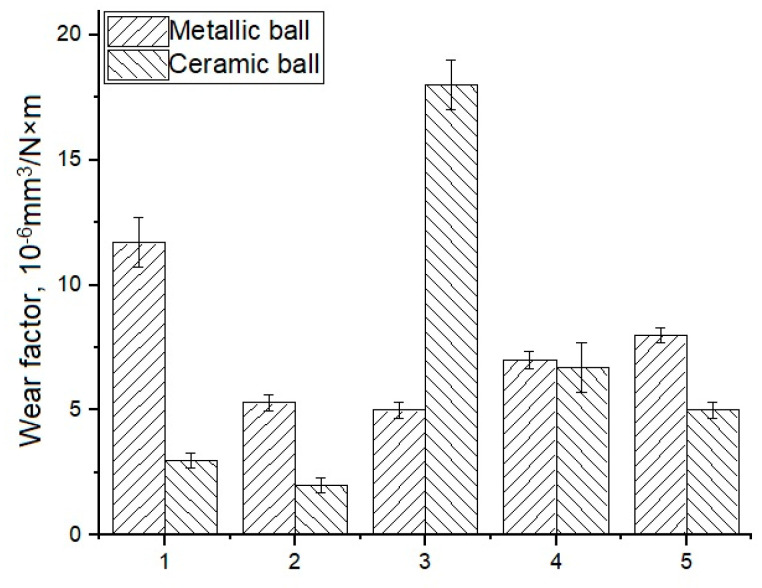
Wear factors of neat PEEK (1) and the PEEK-based composites loaded with 0.3 wt.% nanoparticles—CNF (2); Cu (3); CuFe_2_O_4_ (4), and SiO_2_ (5) during the dry sliding friction on the metal and ceramic counterparts.

A diagram of the wear factor values for neat PEEK and the PEEK-based nanocomposites is shown in Figure 3. When sliding on the steel counterpart, the wear factor of the nanocomposites decreased by 1.5–2.4 times.

When sliding on the ceramic counterpart, the wear factor of all nanocomposites increased by 2–10 times, with the exception of one loaded with CNF (its wear rate decreased by 1.5 times). Accordingly, the studied nanofillers did not play the role of a solid lubricant medium in PEEK (unlike other matrices, for example, thermoplastic semi-crystalline antifriction ultra-high molecular weight polyethylene). In addition, most likely, this pattern of the change in two-component nanocomposites wear resistance was related to both the oxidation processes developing on the friction surfaces and an increase in hardness (as well as their strength, as compared to that of the neat PEEK). Moreover, nanoparticles affected the wearing intensity to varying extents, depending on their chemical nature.

Thus, loading with CNF, copper (Cu), copper ferrite (CuFe_2_O_4_), and silicon dioxide (SiO_2_) nanofillers in small contents (0.3 wt.%) enabled improvement of the strength properties of the PEEK-based composites (elastic modulus increased by 10–15%) through a dispersed hardening mechanism. It was shown that the increase in wear resistance by 1.5–2.3 times in the metal–polymer tribological contact was observed for the composites loaded with 0.3 wt.% CNF and nanoparticles—copper (Cu), silicon dioxide (SiO_2_), and copper ferrite (CuFe_2_O_4_). This was due to the polymer transfer film formation on the steel counterpart, which had enabled protection of the surfaces of both the counterpart itself and the polymer composites from the micro-abrasive damaging and the oxidation.

Loading PEEK with metal nanoparticles resulted in the intensification of the oxidation processes, the counterpart microabrasive wear, and the multiple rising of wear rate in the ceramic-polymer tribological contact. Loading with oxide and ceramic nanoparticles prevented the active development of the oxidation processes, in addition to the formation of the more homogeneous supermolecular structure. This caused the polymer transfer film formation on the counterpart, but could not improve wear resistance compared to that of neat PEEK.

### 3.2. The PEEK-Based Composites Loaded with 7 wt.% Nanoparticles

Since it was not possible to significantly increase the strength and tribological properties of PEEK due to the rather low content of nanoparticles (0.3 wt.%), an attempt was made to enhance the filling degree by several times, similar to that of publications on similar topics (see Table 1). The content of the nanofillers in the PEEK-based composites enabled the improvement of the tribological properties to about 7 wt.%, according to previous studies [44,45]. In order to compare with these data, the results of the studies of the composites loaded with the same nanoparticles, but in an amount of 7 wt.% are presented below.

The physical and mechanical properties of the PEEK-based composites loaded with 7 wt.% nanofillers are shown in Table 4. Their density increased by loading with all types of fillers. Shore D hardness changed in different ways. It decreased by one unit after loading with Cu particles; increased by the same amount after the addition of SiO_2_ particles, and was at the level of neat PEEK, after filling with CNF, as well as CuFe_2_O_4_ particles.

The elastic modulus increased by 3–13%, depending on the filler type. However, it changed insignificantly, compared to the case of loading with 0.3 wt.% of nanofibers and nanoparticles (Table 3). On the other hand, both tensile strength and elongation at break were reduced for all nanocomposites. At the same time, loading with Cu metal nanoparticles caused a decrease in elongation at break to a small extent (dozens percent). Consequently, the tensile strength of Cu nanocomposites also decreased slightly, compared to that of neat PEEK. Loading with carbonaceous CNF, as well as bimetal oxide CuFe_2_O_4_ and ceramic SiO_2_ nanoparticles, on the contrary, resulted in a significant decrease in both elongation at break and tensile strength of the PEEK-based nanocomposites (Table 4). It is highly likely that this effect was associated with the formation of the composite supermolecular structures.

**Figure 4:**
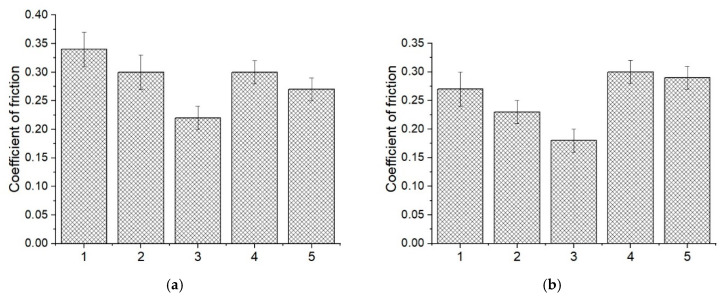
The average values of the friction coefficients for neat PEEK (1) and the PEEK-based nanocomposites loaded with 7 wt. % nanoparticles: CNF (2); Cu (3); CuFe_2_O_4_ (4), and SiO_2_ (5) metal (**a**) and ceramic (**b**) counterparts.

Figure 4 shows the average values of the friction coefficients for neat PEEK and the PEEK-based nanocomposites. When sliding on the metal counterpart (Figure 4a), the friction coefficients of all composites decreased compared to that of neat PEEK. In this case, the lowest value was registered for the composite loaded with Cu nanoparticles (*f* = 0.22 ± 0.02), which was lower by 1.6 times than that of neat PEEK. Other patterns were observed when sliding on the ceramic counterpart. The friction coefficient decreased when loading with CNF, as well as Cu metal nanoparticles, but increased due to loading with SiO_2_ and CuFe_2_O_4_ oxide ones (Figure 4b).

**Figure 5:**
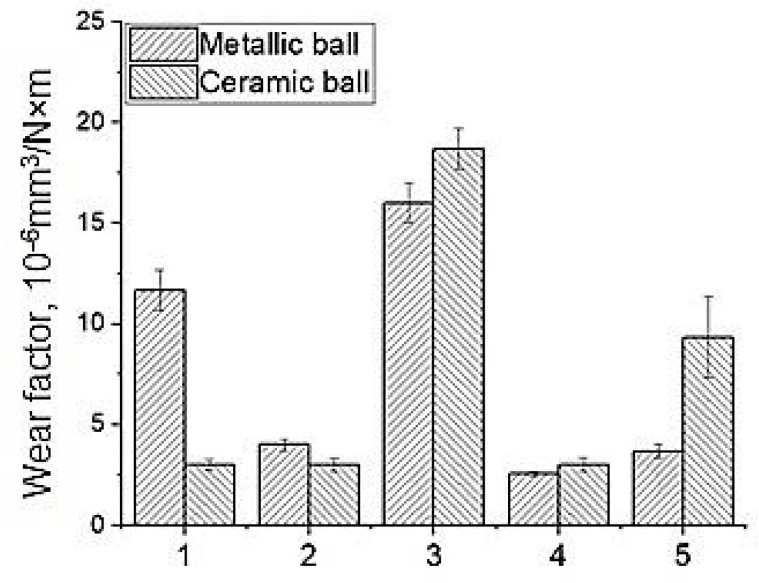
Wear factor of neat PEEK (1) and the PEEK-based composites loaded with 7 wt.% nanoparticles—CNF (2); Cu (3); CuFe_2_O_4_ (4) and SiO_2_ (5) during the dry sliding friction on the metal and ceramic counterparts.

The wear factor diagram for neat PEEK and the PEEK-based composites is shown in Figure 5. When sliding on the metal counterpart, the wear rate decreased by 3.0–4.5 times due to loading with CNF and non-metallic nanoparticles. Filling with metal nanoparticles resulted in an increase in wear factor by up to 1.4 times. Thus, the most efficient fillers for the composites in the metal–polymer tribological contact were CNF, as well as SiO_2_ and CuFe_2_O_4_ ceramic nanoparticles with their content of 7 wt.%. The increase in their content from 0.3 to 7 wt.% caused a decrease in wear rate by 1.3–2.7 times, depending on the type of the fillers (Figure 3 and Figure 5).

When sliding on the ceramic counterpart, a decrease in wear rate was not evident. This parameter values for the composites loaded with CNF and CuFe_2_O_4_ remained at the nearly neat PEEK level. In other cases, the wear rate increased significantly (up to 6 times). Thus, loading with the investigated nanofillers in the amount of 7 wt.% did not enable improvement in wear resistance of the PEEK-based composites for use in the ceramic-polymer tribological contact. 

Protection against the oxidation processes, as well as a solid lubricating effect could be achieved by loading with PTFE particles [46]. For this reason, further wear resistance improvement was supposed to be achieved by simultaneous loading with PTFE solid lubricant particles (including protecting the counterparts from wear by the adhered debris and oxidation products) and nanoparticles. Despite the fact that loading the two-component nanocomposites with 7 wt.% PFTE provided greater increase in wear resistance, SEM micrographs revealed clear signs of nanoparticle agglomeration. This was clearly reflected in the decrease in elongation at break. Since loading with 10 wt.% PTFE also contributed to the deterioration of the supermolecular structure, it was decided to load nanoparticles in the amount of 0.3 wt.% into three-component composites.

### 3.3. Three-Component PEEK-Based Composites Loaded with PTFE and the Nanofillers

This section explores the possibility of further wear resistance improvement by loading with PTFE microparticles in an amount of 10 wt.% and four types of nanofillers in the amount of 0.3 wt.%, namely (i) carbonaceous CNF, (ii) metallic Cu, (iii) bimetal oxide CuFe_2_O_4_, and (iv) ceramic SiO_2_ nanoparticles.

Table 5 presents the physical and mechanical properties of the PEEK-based composites loaded with 10 wt.% PTFE and 0.3 wt.% nanofillers. It could be concluded that:

(1) Density of the three-component composites increased by loading with the nanofillers.
(2) Their Shore D hardness decreased by 3–4 units compared to that of neat PEEK and was at the level of the “PEEK/10PTFE” composite.
(3) The elastic modulus decreased slightly (down to 10%) by loading with CNF, Cu, and SiO_2_, and increased by filling with CuFe_2_O_4_ (up to 10%), as compared to that of the “PEEK/10PTFE” composite.
(4) Tensile strength increased by loading with the nanofillers—the “PEEK/10PTFE/0.3CuFe_2_O_4_” composite possessed the maximum value of 95.5 MPa, which was 12 MPa higher, compared to that of the “PEEK/10PTFE” composite.
5) The values of elongation at break also increased by Δε = 3–5%, due to loading with the nanofillers compared to that of the “PEEK/10PTFE” composite.

Thus, increasing the elastic modulus of the three-component composites by loading with the nanofillers was not evident, with the exception of CuFe_2_O_4_ nanoparticles.

**Figure 6:**
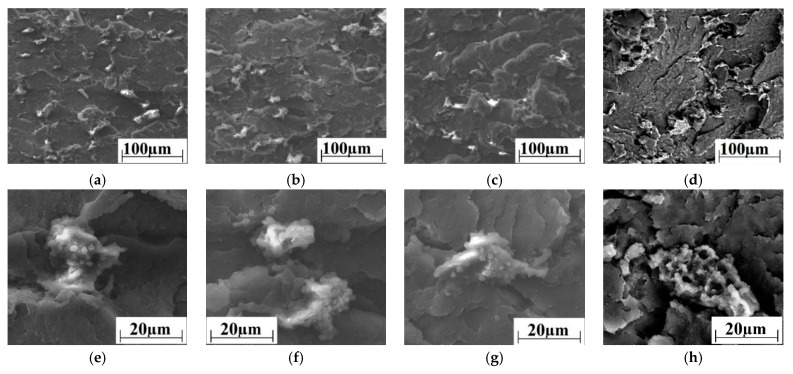
The SEM micrographs of the supermolecular structure of the PEEK-based composites: “PEEK/10PTFE/0.3CNF” (**a**,**e**); “PEEK/10PTFE/0.3Cu” (**b**,**f**); “PEEK/10PTFE/0.3CuFe_2_O_4_“ (**c**,**g**); and “PEEK/10PTFE/0.3SiO_2_“ (**d**,**h**).

Figure 6 illustrates the supermolecular structure of the studied three-component composites. PTFE particles were distributed quasi-uniformly over the boundaries of the structural elements of the polymer supermolecular structure (Figure 6a–d). It could be seen at higher magnification (Figure 6e–h) that the nanofillers were segregated and agglomerated in the region of PTFE inclusions. Apparently, due to this fact, the dispersed hardening effect was not pronounced in the three-component composites loaded with the nanofillers, unlike the case of the two-component ones.

**Figure 7:**
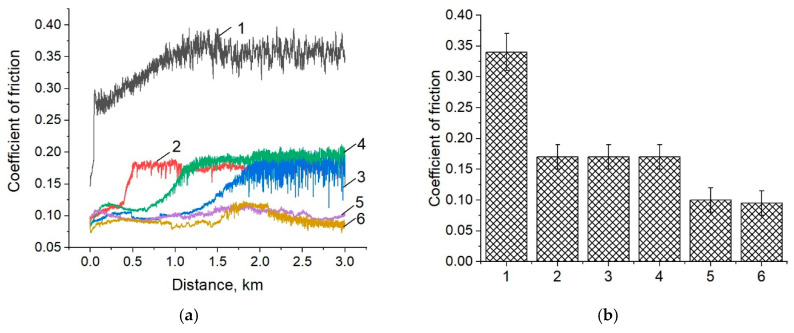

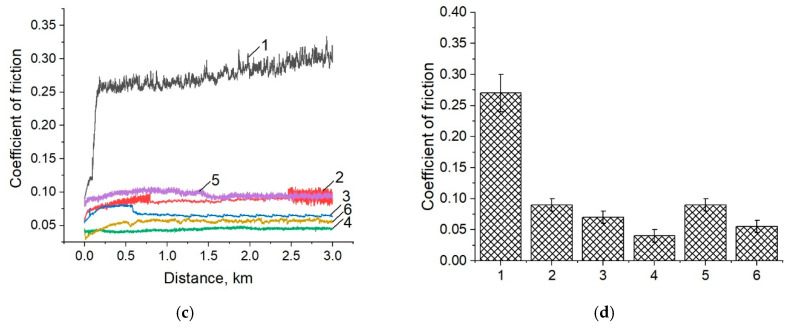
The dependences of the friction coefficients versus the sliding distance (**a**,**c**), as well as their average values (**b**,**d**) for neat PEEK (1) and the PEEK-based composites: “PEEK/10PTFE” (2); “PEEK/10PTFE/0.3CNF” (3); “PEEK/10PTFE/0.3Cu” (4); “PEEK/10PTFE/0.3CuFe_2_O_4_” (5), and “PEEK/10PTFE/0.3SiO_2_“ (6); metal (**a**,**b**) and ceramic (**c**,**d**) counterparts.

Figure 7 shows the dependences of the friction coefficients versus the sliding distance, as well as their average values for neat PEEK and the three-component PEEK-based composites. The friction coefficients of the three-component composites loaded with CNF and copper gradually increased, when sliding on the metal counterpart at a distance of up to 1.5 km. Then it reached a constant value and was at the level of the “PEEK/10PTFE” one (f ≈ 0.15–0.17; Figure 7, curves 2, 3, and 4). However, the minimum value of the friction coefficient was 0.1 for the “PEEK/10PTFE/0.3CuFe_2_O_4_” and “PEEK/10PTFE/0.3SiO_2_” composites, which was 1.7 times lowerthan that of the “PEEK/10PTFE” one, while it was lower by 3.4 times than that of the neat PEEK. Noteworthy, they remained constant over the entire testing range.

Other patterns were observed when sliding on the ceramic counterpart. The friction coefficients varied slightly for all investigated three-component nanocomposites over the entire sliding distance (Figure 7c) and did not exceed 0.1. Its lowest value 0.04 was observed for the “PEEK/10PTFE/0.3Cu” composite, which was two times lower than that for the “PEEK/10PTFE” one, and seven times lower than that for neat PEEK (Figure 7d).

**Figure 8:**
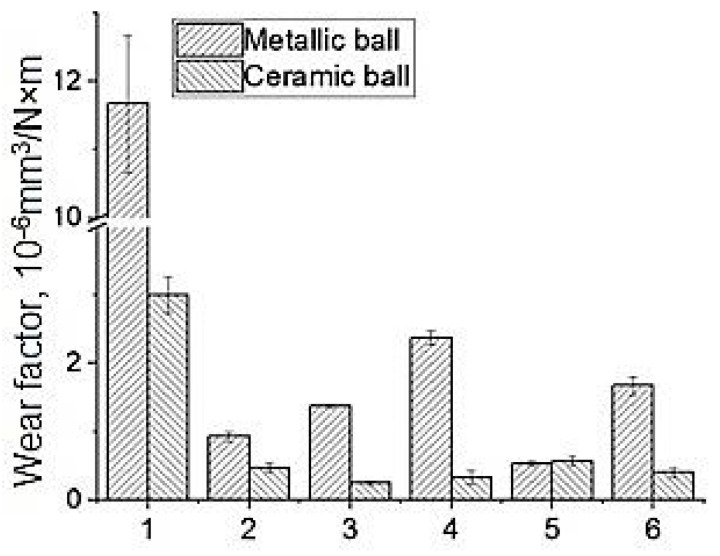
Wear rates of neat PEEK (1) and the PEEK-based composites: “PEEK/10PTFE” (2); “PEEK/10PTFE/0.3CNF” (3); “PEEK/10PTFE/0.3Cu” (4); “PEEK/10PTFE/0.3CuFe_2_O_4_” (5) and “PEEK/10PTFE/0.3SiO_2_“ (6), under dry sliding friction conditions on the metal and ceramic counterparts.

The wear rate diagram for neat PEEK and the three-component PEEK-based nanocomposites is shown in Figure 8. When sliding on the steel counterpart, the lowest wear rate was registered for the “PEEK/10PTFE/0.3CuFe_2_O_4_” composite. Wear factor was 0.53 ± 0.033·10^−6^ mm^3^/N·m, which was 1.75 times less than that for the “PEEK/10PTFE” composite, and 22 times less than that for neat PEEK. Thus, the greatest increase in wear resistance for the metal–polymer tribological contact under the dry sliding friction conditions was achieved for the three-component PEEK-based composites loaded with 0.3 wt.% CuFe_2_O_4_ bimetal oxide nanoparticles.

However, the lowest wear factor on the ceramic counterpart was observed for the “PEEK/10PTFE/0.3CNF” composite. Wear factor was 0.25 ± 0.033·10^−6^ mm^3^/N·m, which was two times less than that for the “PEEK/10PTFE” composite and 12 times less than that for neat PEEK. However, it was very close to the “PEEK/10PTFE/0.3Cu”; with a wear factor of 0.28 ± 0.065·10^−6^ mm^3^/N·m. When sliding on the harder ceramic counterpart, the wear resistance improvement was primarily associated with the transfer of film formation and adherence on its surface. This protected the polymer from the oxidation and periodic impacts of the sliding ceramic ball, as well as the ceramic counterpart from the abrasive debris wear (discussed in detail below in the analysis of the wear track surfaces of both the composites and the counterpart). In this case, the effect of nanoparticles should be discussed from the standpoint of the transfer film adhesion on the counterpart surface.

**Figure 9:**
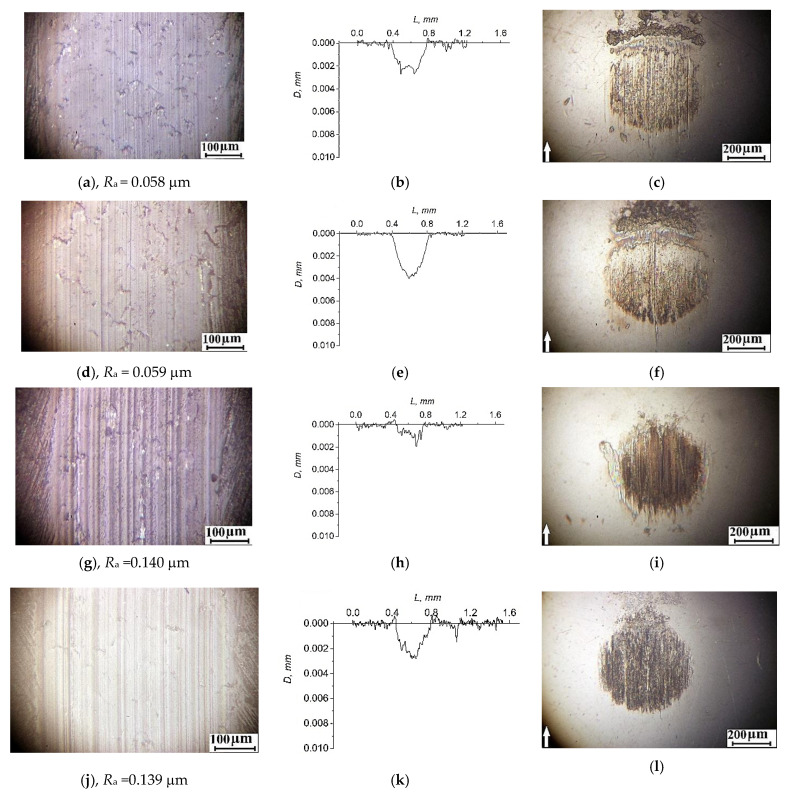
The topography of the wear track surfaces of the polymer samples and the wear scar surfaces of steel counterparts, as well as the wear track profiles (the sliding distance of 3 km)—the “PEEK/10PTFE/0.3CNF” (**a**–**c**); “PEEK/10PTFE/0.3Cu” (**d**–**f**); “PEEK/10PTFE/0.3CuFe_2_O_4_” (**g**–**i**); and “PEEK/10PTFE/0.3SiO_2_“ (**j**–**l**) composites.

**Figure 10:**
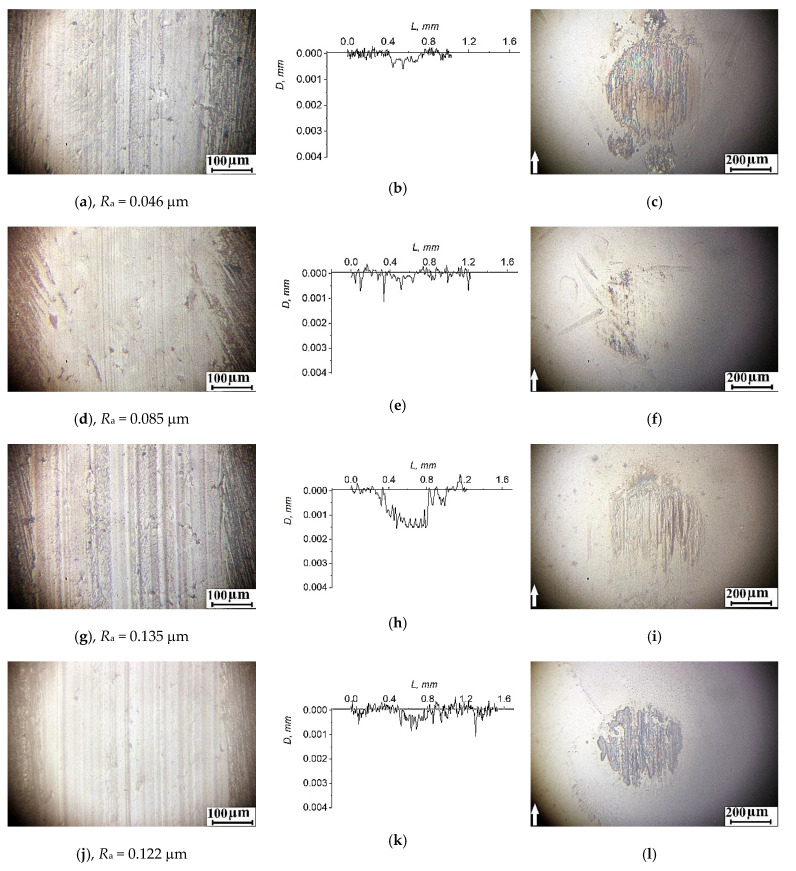
The topography of the wear track surfaces of the polymer samples and the wear scar surfaces of ceramic counterparts, as well as the wear track profiles (the sliding distance of 3 km)—the “PEEK/10PTFE/0.3CNF” (**a**–**c**); “PEEK/10PTFE/0.3Cu” (**d**–**f**); “PEEK/10PTFE/0.3CuFe_2_O_4_” (**g**–**i**); and “PEEK/10PTFE/0.3SiO_2_“ (**j**–**l**) composites.

Figure 9 and Figure 10 show the optical micrographs characterizing the topography of the wear track surfaces of the studied three-component composites, the steel and ceramic counterpart, as well as the wear track profiles.

In the metal–polymer tribological contact, a similar pattern of the topography of the wear track surfaces was observed for the “PEEK/10PTFE/0.3CNF” and “PEEK/10PTFE/0.3Cu” composites. Small shallow longitudinal micro-grooves formed on the sliding surfaces of the composites (Figure 9a,d). Roughness of the composite surfaces was low and almost the same (0.058 and 0.059 μm). On the steel counterpart surface, individual small longitudinal micro-grooves were also visible, while a thin transfer film had also formed (Figure 9c,f). There were almost no damage traces (micro-scratching) of the counterparts.

Wear rate of the “PEEK/10PTFE/0.3CuFe_2_O_4_” composite was lower than that of the other three-component ones, although micro-grooves on its surface were deeper (Figure 9g). This was confirmed by the roughness measurement data, which increased by almost three times by up to 0.140 μm. Despite this fact, the metal counterpart surface was also protected by a highly oxidized polymer transfer film (Figure 9i). The most probable reason for the formation of such micro-grooves was the micro-abrasive debris damage with the dispersion-hardened composite. It should be noted that the transfer film was securely fixed on the friction surface of the steel counterpart. In general, a similar pattern was observed for the “PEEK/10PTFE/0.3SiO_2_“composite, although the wear rate was almost twice as high in this case.

In the ceramic-polymer tribological contact, the “PEEK/10PTFE/0.3CNF” composite possessed the lowest wear intensity. There were almost no longitudinal micro-grooves on its wear track surface. Roughness of 0.046 μm was very low (Figure 10a). A transfer film was adhered on the counterpart surface, which was similar to the “PEEK/10PTFE” composite.

A close value of wear factor and a similar wear pattern was typical for the “PEEK/10PTFE/0.3Cu” composite. The smooth wear surface with a low roughness of 0.085 μm was also observed (Figure 10d). A transfer film was almost not visible on the ceramic counterpart surface (Figure 10f).

In contrast, longitudinal micro-grooves were on the surfaces of both “PEEK/10PTFE/0.3CuFe_2_O_4_” and “PEEK/10PTFE/0.3SiO_2_“composite wear tracks (Figure 10g,h,j,k) and the counterparts (Figure 10i,l). Roughness increased up to 0.122–0.135 μm. At the same time, wear factor doubled in comparison with that of the “PEEK/10PTFE/0.3CNF” composite. Wear debris was found adhered on the ceramic counterpart surface (Figure 10i,l), which protected both the counterpart and the polymer composite from intensive wearing.

Thus, when sliding on the metal counterpart, the “PEEK/10PTFE/0.3CuFe_2_O_4_” composite possessed wear resistance, which improved by 1.75 times compared to that of “PEEK/10PTFE” (which was 22 times higher than that of neat PEEK). When sliding on the ceramic counterpart, the lowest wear was achieved for the “PEEK/10PTFE/0.3CNF” composite. Its wear resistance was two times higher than that of “PEEK/10PTFE” (which was 12 times higher than that of neat PEEK). However, it was very close to “PEEK/10PTFE/0.3Cu” one.

**Figure 11:**
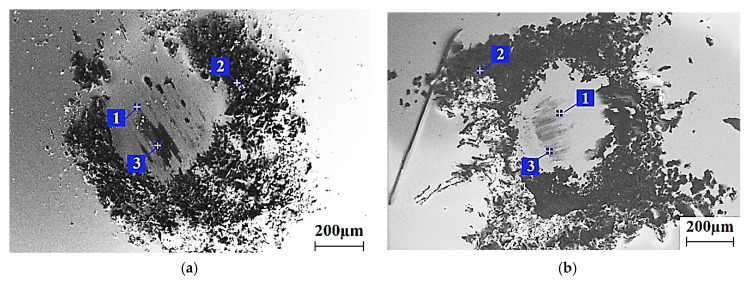
SEM micrographs of the steel (**a**) and ceramic (**b**) counterpart surfaces after the tribological tests.

Since the increase in wear resistance was determined by the formation and the adhesion of the transfer film to the counterpart surface, further analysis was carried out for three-component nanocomposites loaded with PTFE. Their microanalysis (EDS) was carried out for the both metal- and ceramic-polymer tribological contacts, after loading with the metal (Cu) and ceramic (CuFe_2_O_4_) nanofillers. They possessed nearly the highest wear resistance, while their volumetric content and specific surface area were approximately equivalent (Table 2). Figure 11 shows the SEM micrographs of the surfaces of the metal and ceramic counterparts after the tribological tests. The chemical composition of the transfer film is presented in Table 6.

The data in Figure 11 indicate that the Cu (metal) and CuFe_2_O_4_ (bimetal oxide) nanofillers, in addition to the PTFE, formed the transfer film on the counterparts and adhered to their surface (the spectra at point 3). The spectra at point 2 corresponded to debris that did not adhered to the surface in the form of a thin film. It was characterized by an increased content of PTFE (Table 6). It should be noted that the data on the oxygen content were consciously excluded, since its presence could be associated not only with the tribological polymer oxidation, but also followed from its presence in the SEM chamber.

A more pronounced (contrasting) transfer film formed from the nanofiller (Figure 11a) on the metal counterpart, compared to the ceramic one (Figure 11b). This was due to the higher activity of the counterpart material (steel compared to ceramics), which determined the higher wear resistance of the metal–polymer contact (Figure 9 and Figure 10). Due to the predominant agglomeration of nanoparticles within PTFE inclusions, they were easier separated and transferred to the counterpart surface. Then, the transfer film was fixed on the counterpart surface due to the PFTE tribological oxidation, which resulted in the improved wear resistance, as compared to the “PEEK + 10 wt.% PFTE” composite.

Different types of nanoparticles affected wear resistance in many ways. due to the following reasons—(i) various specific surface area; (ii) distinctive agglomeration degrees (for example, according to the manufacturer’s data, the initial Cu nanoparticles were highly agglomerated compared to CuFe_2_O_4_); (iii) divergent volumetric contents (and not the same agglomeration degrees as a result); (iv) unequable chemical activity; and (v) miscellaneous specific surface. Accordingly, nanoparticles in the transfer film on the counterpart surface determined the duration of the “polymer-transfer film” conditions in the tribological contact, providing the low stable friction coefficient values and improved wear resistance.

It should be noted as a conclusion that the obtained results showed the dual function of nanoparticles in the “PEEK/PFTE/nanofiller” composite—(i) adhesion of the transfer film to the counterpart, and (ii) dispersed hardening, which increased the deformation and strength properties (tensile strength, elongation at break), as compared to the “PEEK/PTFE” composite. The nanofiller type (its composition) determined the tribological oxidation level and, as a consequence, the formation and retention of the transfer film on the counterpart. The volumetric content of the nanofillers affected their ability to form a transfer film on the counterpart, to a much lesser extent, both in the metal- and ceramic-polymer tribological contacts. However, it could facilitate the separation of debris from the friction surface of the composite, characterized by the presence of the agglomerated nanofiller.

### 3.4. Interpritation of Results

In this research, the aim of loading the PEEK-based composites with the nanofiller was to improve their tribological characteristics due to the formation of adhesive layers on the counterpart and, as a result, to fix the transfer film that contained the “PEEK/PTFE” debris (but not to enhance their physical and mechanical properties, since it is now undeniable that improving wear resistance while maintaining the strength characteristics at the level of the neat polymer is the main challenge of designing advanced PEEK-based materials). The studied nanofillers did not play the role of a solid lubricating medium in the composites (unlike other matrices, for example, thermoplastic semi-crystalline antifriction UHMWPE). After loading, this character of change in wear resistance was likely based on both the tribological oxidation processes developed on the wear surfaces and the increase in their hardness. In this case, nanoparticles affected the intensity to varying degrees, depending on their chemical nature. The achieved low wear resistance values (in comparison to that of neat PEEK), the observed high wear of the metal and ceramic counterparts, as well as the noticeable decrease in the mechanical properties, enabled us to conclude that the loading level of 7 wt.% was too high for the studied nanoparticles.

**Figure 12:**
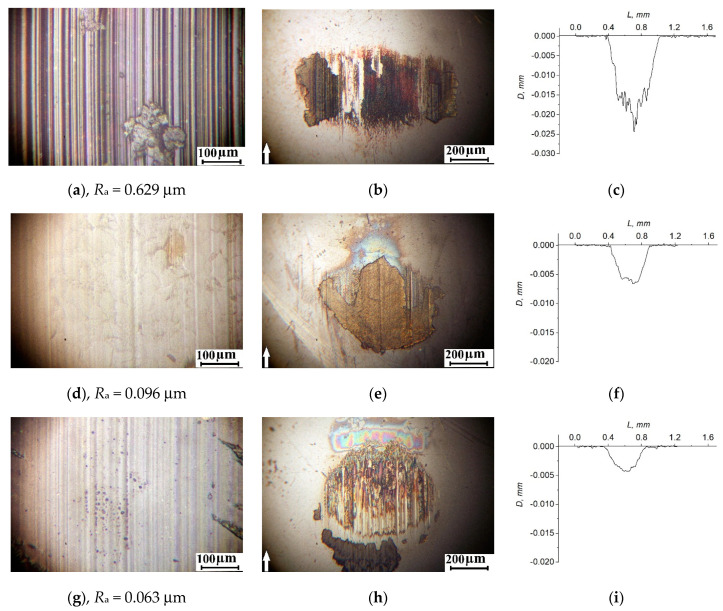
The topography of the wear surfaces on the polymer composites and the steel counterpart, as well as wear track profiles after a test distance of 3 km— “PEEK/7Cu” (**a**–**c**); “PEEK/7SiO_2_” (**d**–**f**); and “PEEK/7CuFe_2_O_4_” (**g**–**i**).

Figure 12 shows optical micrographs characterizing the topography of the wear surfaces of all studied composites on the steel counterpart, as well as the wear track profiles. Deep longitudinal micro-grooves were observed on the wear surfaces of the “PEEK/7Cu” composites, loaded with metal nanoparticles (Figure 12a,b). At the same time, roughness increased by several times, up to Ra = 0.6 μm. There was a lot of debris on the counterpart surface, due to the tribological oxidation of copper and polymer (Figure 12b). The micro-abrasive effect of these solid particles (they had actually plowed, as evidenced by the wear track profilograms with substantially non-smooth “ragged” profiles, Figure 12c) was the main reason for the multiple increase in the wear rate values.

For the “PEEK/7SiO_2_“ and “PEEK/7CuFe_2_O_4_” composites, the formed thin transfer films (of the rainbow glow) were observed on the steel counterpart surface (Figure 12e,h). They protected the counterpart and the composites from intense micro-abrasion wear. It should be noted that a structural inhomogeneity associated with excessive PEEK filling was found on the wear surface of the “PEEK/7SiO_2_“composite (Figure 12e). For this reason, the debris fixation on the counterpart surface was observed in the form of irregularly shaped formations (Figure 12e).

**Figure 13:**
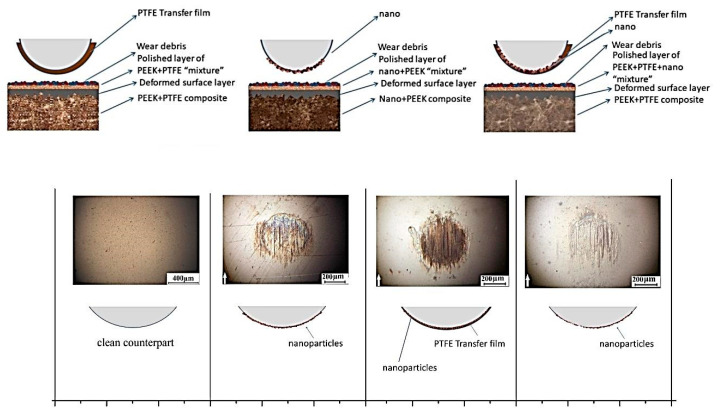
The scheme of the transfer film formation for the “PEEK/PTFE” (1), “PEEK/nano” (2), and “PEEK/PTFE/nano” (3) composites.

On the other hand, protection against the tribological oxidation processes and providing the solid lubricating effect was achieved by the PEEK loading with PTFE particles. It was proven that the mechanism of the lubricating action of the neat PTFE and PTFE-based composites was similar [47], due to the specific fluoroplastic structure. The PTFE transfer film was not continuous, due to poor adhesion. In addition, it consisted of separate fragments, which were removed from the friction zone when the critical thickness of 10–40 nm was reached [48]. Wear of the antifriction composites that contained fluoroplastic occurred according to the adhesive mechanism, the basis of which was both the frictional transfer and the weak adhesive interaction with the counterparts. The low friction coefficient value was provided by the specific supermolecular fluoroplastic structure, as well as the mobility of its molecular chains, and as a result, there was a low resistance to deformation and poor adhesion to the counterparts [49]. Figure 13 shows a scheme of the transfer film formation for the PEEK-based composites.

**Figure 14:**
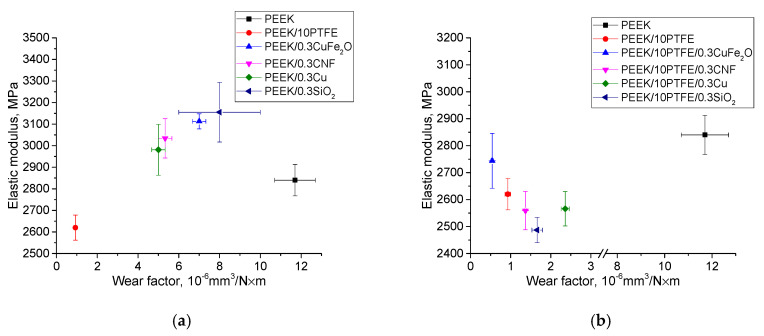

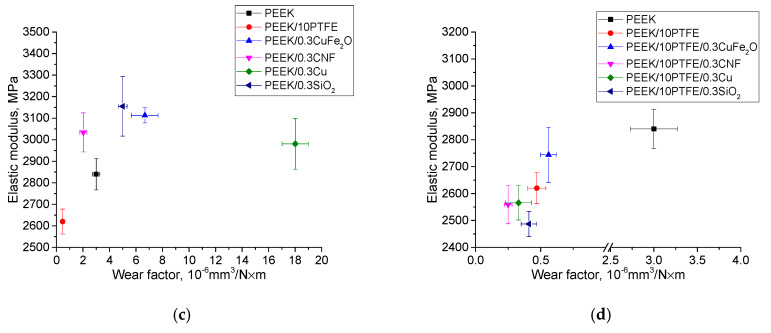
The elastic modulus values vs. wear factor on the metal (**a**,**b**) and ceramic (**c**,**d**) counterparts.

Figure 14 shows the dependence of the elastic modulus values from the wear rates for neat PEEK, as well as for the two- and three-component PEEK-based composites.

**Figure 15:**
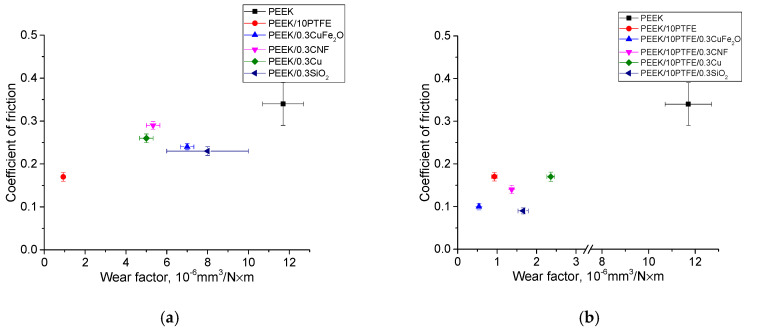

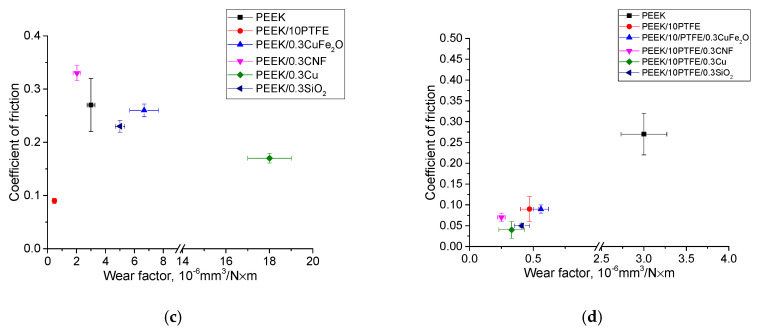
The friction coefficients vs. wear factor on the metal (**a**,**b**) and ceramic (**c**,**d**) counterparts.

Figure 15 shows the dependence of the friction coefficients values from the wear rates for neat PEEK, as well as for the two- and three-component PEEK-based composites.

## 4. Conclusions

The mechanical and tribological properties of the composites, based on PEEK and PEEK+PTFE, with various types of nanofillers (carbonaceous, metallic, bimetal oxide, ceramic), under the conditions of the metal- and ceramic-polymer tribological contacts, were investigated. Based on the obtained results, the following conclusions were drawn.

1. It was shown that loading with CNF, Cu, SiO_2_, and CuFe_2_O_4_ nanoparticles in the small content (0.3 wt.%) enabled improvement of the elastic modulus of the PEEK-based composites by 10–15%. Wear resistance of the composites loaded with 0.3 wt.% of the nanofillers increased by 1.5–2.3 times in the metal–polymer tribological contact. This was due to the polymer transfer film formation on the steel counterpart. In the ceramic–polymer tribological contact, loading PEEK with metal nanoparticles caused the intensification of the oxidation processes, the abrasive counterpart wear, and the multiple increases in wear rate. This was accompanied by the polymer transfer film formation on the counterpart, but was not able to improve the wear resistance compared to that of neat PEEK.
2. The formation of the transfer film from the PEEK-based nanocomposite debris on the steel counterpart surface was determined by its supermolecular structure, which is susceptible to destruction, while the ability to fix it depended on the activity of nanoparticles. In the case of CNF and Cu, the transfer film on the counterpart was less oxidized, which reduced the wear rate of the polymer composite. In the tribological tests of the PEEK-based composites loaded with SiO_2_ and CuFe_2_O_4_ nanoparticles, the transfer film was more oxidized. This caused more intense damages and wear of the polymer nanocomposite friction surface.
3. The three-component PEEK-based composites loaded with PTFE and nanoparticles, with the slight decrease in the mechanical properties, provided an increase in wear resistance under the dry sliding friction conditions by up to 22 times in the metal–polymer tribological contact, and up to 12 times (at the wear-free level) in the ceramic-polymer one, compared to that of the neat PEEK. In all cases, this was achieved by the PTFE containing transfer film formation and adhering to the counterpart.
4. The dispersed hardening effect was not pronounced in the three-component PEEK-based composites loaded with nanofillers, unlike the case of the two-component ones. Due to the predominant agglomeration of nanoparticles within PTFE inclusions, they were easier separated and transferred to the counterpart surface. Then, the transfer film adhered on the counterpart surface due to the PEEK tribological oxidation resulted in improved wear resistance, compared to the “PEEK/10PTFE” composite.
5. In the "PEEK/10PTFE/0.3 nanofiller” composite, the nanoparticles served a dual function—(i) adhesion of the transfer film to the counterpart and (ii) dispersed hardening, which increased the deformation and strength properties (tensile strength, elongation), as compared to the “PEEK/10PTFE” composite. The nanofiller type (its composition) determined the tribological oxidation level and, as a consequence, also determined the formation and adherence of the transfer film on the counterpart.

## Figures and Tables

**Table 1:** Wear resistance of some polymer nanocomposites [35].

Matrix	Type of Nanoparticles (Particle Size)	The Lowest Wear Rate (10^–6^mm^3^/N·m)	The Optimum Content of Nanoparticles (vol./wt.%)
PEEK	Si_3_N_4_ (<50 nm)	1.3	2.8/7.5
PEEK	SiO_2_ (<100 nm)	1.4	3.4/7.5
PEEK	SiC (80 nm)	3.4	1–3/2.5–10
PEEK	ZrO_2_ (10 nm)	3.9	1.5/7.5

**Table 2:** Parameters of the nanofillers used.

Nanofiller Type	Content (wt/vol.%)	Specific Area, m^2^/g	Specific Heat Conductivity Wt/cm⋅K
CNF	0.3/0.24	≥160	4.5–10
Cu	0.3/0.044	8.0 ± 0.5	400
CuFe_2_O_4_	0.3/0.076	14.2 ± 0.5	1.8–2.0
SiO_2_	0.3/0.149	≥130	1.3–1.5

**Table 3:** The physical and mechanical properties of neat PEEK and the PEEK-based composites loaded with 0.3 wt.% nanoparticles.

Filler Content, wt. %	Density *ρ*, g/cm^3^	Shore D Hardness	Elastic Modulus *E*, MPa	Tensile Strength *σ*, MPa	Elongation at Break *ε*, %
PEEK	1.308	80.1 ± 1.17	2840 ± 273	106.9 ± 4.7	25.6 ± 7.2
PEEK/0.3CNF	1.314	80.3 ± 0.2	3034 ± 91	107.8 ± 1.7	23.6 ± 4.3
PEEK/0.3Cu	1.324	79.5 ± 0.5	2981 ± 118	100.9 ± 4.4	17.2 ± 3.9
PEEK/0.3CuFe_2_O_4_	1.309	80.2 ± 0.9	3113 ± 35	108.4 ± 0.4	19.8 ± 1.3
PEEK/0.3%SiO_2_	1.317	81.0 ± 0.6	3155 ± 238	111.4 ± 1.6	14.7 ± 3.2

**Table 4:** The physical and mechanical properties of the PEEK-based composites loaded with 7 wt.% nanoparticles.

Filler Type and Content, wt.%	Density *ρ*, g/cm^3^	Shore D Hardness	Elastic Modulus *E*, MPa	Tensile Strength *σ*, MPa	Elongation at Break *ε*, %
PEEK	1.308	80.1 ± 1.17	2840 ± 273	106.9 ± 4.7	25.6 ± 7.2
PEEK/7CNF	1.344	79.9 ± 0.4	3191 ± 43	91.3 ± 12.6	3.6 ± 0.6
PEEK/7Cu	1.375	78.9 ± 0.4	2937 ± 199	104.4 ± 1.8	14.0 ± 3.9
PEEK/7SiO_2_	1.354	81.4 ± 0.3	2860 ± 90	83.5 ± 20.5	4.0 ± 1.5
PEEK/7CuFe_2_O_4_	1.370	80.2 ± 0.9	3058 ± 257	102.4 ± 5.3	6.3 ± 1.7

**Table 5:** The physical and mechanical properties of the PEEK-based composites loaded with 10 wt.% PTFE and 0.3 wt.% nanoparticles.

Filler Content, wt.%	Density *ρ*, g/cm^3^	Shore D Hardness	Elastic Modulus *E*, MPa	Tensile Strength *σ*, MPa	Elongation at Break *ε*, %
PEEK	1.308	80.1 ± 1.17	2840 ± 273	106.9 ± 4.7	25.6 ± 7.2
PEEK/10PTFE	1.324	77.3 ± 0.24	2620 ± 158	83.9 ± 2.4	5.0 ± 0.8
PEEK/10PTFE/0.3CNF	1.344	77.2 ± 0.3	2559 ± 71	86.4 ± 0.6	8.2 ± 1.7
PEEK/10PTFE/0.3Cu	1.356	77.9 ± 0.3	2566 ± 64	88.6 ± 0.9	10.1 ± 2.3
PEEK/10PTFE/0.3CuFe_2_O_4_	1.352	77.6 ± 0.2	2744 ± 102	95.5 ± 4.1	8.2 ± 1.1
PEEK/10PTFE/0.3SiO_2_	1.341	76.6 ± 0.2	2487 ± 47	91.8 ± 2.4	7.8 ± 1.2

**Table 6:** The results of EDS analysis of the transfer film and debris on the steel and ceramic counterparts in accordance with the labels in Figure 11.

Element	Spectrum 1 wt%/ at.%	Spectrum 2 wt%/ at.%	Spectrum 3 wt%/ at.%
**Steel Counterpart**			
Cr	1.72/1.84	-	1.31/0.58
Fe	98.28/98.16	0.69/0.17	58.57/23.76
C	-	75.19/83.05	40.08/75.64
F	-	23.87/16.66	-
Si	-	0.25/0.12	-
Cu	-	-	0.04/0.12
**Ceramic Counterpart**			
Zr	26.37/4.51	-	-
Fe	-	-	-
C	73.63/95.49-	67.52/76.68	99.35/99.88
F	-	32.48/23.32	-
Si	-	0.25/0.12	-
Cu	-	-	0.65/0.12

## Data Availability

The data presented in this study are available on request from the corresponding author.
